# Testing Mayo Clinic’s New 20/20/20 Risk Model in Another Cohort of Smoldering Myeloma Patients: A Retrospective Study

**DOI:** 10.3390/curroncol28030188

**Published:** 2021-05-26

**Authors:** Camille Tessier, Thomas Allard, Jean-Samuel Boudreault, Rayan Kaedbey, Vincent Éthier, Fléchère Fortin, Michel Pavic

**Affiliations:** 1 Centre Hospitalier Universitaire de Sherbrooke (CHUS), Sherbrooke, QC J1H 5H3, Canada; camille.tessier@usherbrooke.ca (C.T.); Thomas.Allard@USherbrooke.ca (T.A.); Vincent.Ethier@USherbrooke.ca (V.É.); Flechere.Fortin@USherbrooke.ca (F.F.); 2 Hôpital Universitaire du Sacré-Cœur de Montréal, Montreal, QC H4J 1C5, Canada; jean-samuel.boudreault-pedneault@umontreal.ca; 3 Jewish General Hospital, McGill University, Montreal, QC H3T 1E2, Canada; rayan.kaedbey@mcgill.ca; 4 Centre de Recherche du CHUS (CRCHUS), Sherbrooke, QC J1H 5N4, Canada

**Keywords:** smoldering multiple myeloma, multiple myeloma, risk stratification model

## Abstract

Background—smoldering multiple myeloma (SMM) risk of progression to multiple myeloma (MM) is highly heterogeneous and several models have been suggested to predict this risk. Lakshman et al. recently proposed a model based on three biomarkers: bone marrow plasma cell (BMPC) percentage > 20%, free light chain ratio (FLCr) > 20 and serum M protein > 20 g/L. The goal of our study was to test this “20/20/20” model in our population and to determine if similar results could be obtained in another cohort of SMM patients. Method—we conducted a retrospective, single center study with 89 patients diagnosed with SMM between January 2008 and December 2019. Results—all three tested biomarkers were associated with an increased risk of progression: BMPC percentage ≥ 20% (hazard ratio [HR]: 4.28 [95%C.I., 1.90–9.61]; *p* < 0.001), serum M protein ≥ 20 g/L (HR: 4.20 [95%C.I., 1.90–15.53]; *p* = 0.032) and FLCr ≥ 20 (HR: 3.25 [95%C.I., 1.09–9.71]; *p* = 0.035). The estimated median time to progression (TTP) was not reached for the low and intermediate risk groups and was 29.1 months (95%C.I., 3.9–54.4) in the high-risk group (*p* = 0.006). Conclusions—the 20/20/20 risk stratification model adequately predicted progression in our population and is easy to use in various clinical settings.

## 1. Introduction

First described in 1980 by Kyle and Greipp [1], smoldering multiple myeloma (SMM) is a rare monoclonal plasma cell disorder, affecting 0.4–0.9 persons per 100,000 [2,3]. Although infrequent, SMM is clinically significant because approximately 75% of patients will eventually progress to symptomatic multiple myeloma (MM) [4], a hematologic malignancy associated with poor prognosis. As its name implies, SMM distinguishes itself from MM by the absence of symptoms, i.e., the absence of CRAB features (hypercalcemia, renal failure, anemia or bone lesion). However, even in the absence of symptoms, a diagnosis of MM can be reached if certain specific biomarkers are present (namely, bone marrow plasma cell (BMPC) > 60%, serum free light chain ratio (FLCr) > 100 or >1 lesion of 5 mm on skeletal magnetic resonance imaging (MRI)) [5]. These biomarkers were added to the diagnostic criteria of MM a few years ago because several cohort analyses have shown that their presence is associated with an 80–90% risk of progression at 2 years [5,6,7].

Currently, the standard of care for SMM is watchful waiting until the emergence of symptoms or myeloma biomarkers. This approach comes from studies that did not found any advantage in progression-free survival (PFS) or overall survival (OS) by starting treatments at diagnosis [8,9,10]. However, in recent studies selecting only patients at high risk of progression, early treatments were associated with survival benefits [11,12,13]. There is, therefore, a growing interest in identifying high risk patients to explore therapeutic options in this specific subgroup. Many efforts have been made, throughout the last years, to develop a risk stratification model able to predict the risk of progression of SMM, however, none has yet been adopted internationally.

In 2018, after a large cohort study, the Mayo Clinic group proposed a model based on three risk factors: BMPC > 20%, serum M-protein > 20 g/L and FLCr > 20 [14]. In this “20/20/20” model, patients with 0, 1 or ≥2 risk factors are respectively considered at low, intermediate and high-risk of progression. More recently, it has been suggested to include high-risk cytogenetic abnormalities as a fourth risk factor to this model, to further improve its accuracy [15]. This alternative version of the 20/20/20 model arose from another large cohort study which reported that certain cytogenetic abnormalities (i.e., translocation t(4;14), t(14;16), 1q gain, del13q and monosomy 13) are an independent risk factor for progression [15]. To take into account this fourth risk factor, this alternative version of the 20/20/20 model includes a fourth risk category and patients with 0, 1, 2 or ≥3 risk factors are respectively considered at low, intermediate-low, intermediate and high risk of progression. Reported PFS at 2 years for both version of the model is presented in Table 1.

The 20/20/20 risk stratification model proposed by Lakshman et al. shows promising results and is appealing because the risk factors involved are commonly determined at diagnosis, making its applicability universal. However, before being widely adopted, this model needs to be tested in a variety of clinical settings to determine its accuracy and reproducibility. Therefore, the primary goal of our study is to test this new stratification model in our population and to determine if it could adequately predict time to progression (TTP) in each risk group. In addition to the three markers used in the 20/20/20 model, we also tested the impact on progression of various demographic and biochemical markers such as age, immunoparesis, and high levels of β_2_-microglobulin.

## 2. Materials and Methods

### 2.1. Patients

We conducted a retrospective study of patients with SMM seen at the Centre hospitalier de l’Université de Sherbrooke (CHUS) between January 2008 and December 2019. Patients were identified by query of the electronic medical records and the 2014 International Myeloma Working Group (IMWG) updated diagnostic criteria for SMM were used to determine eligibility [5]. Patients were excluded if they had received any form of chemotherapy for another cancer after their diagnosis of SMM or had ≤2 years follow-up. The data cut-off date was 1 January 2020. Approval of this study was obtained from the Research Ethics Board of the CHUS on 7 January 2020 (Project Approval Number 2020–3487).

### 2.2. Interphase Fluorescent In Situ Hybridization

BMPCs were isolated using anti-CD138 immunomagnetic beads (EasySep™ Human CD138 Positive Selection Kit II, Stemcell Technologies, Vancouver, BC, Canada). Interphase fluorescent in situ hybridization (iFISH) analysis was performed on CD138-purified BMPCs using the following probes: 17p13.1 (TP53), 14q32 (IGH), 4p16.3 (FGFR3), 16q23 (MAF), 20q12 (MAFB), 1q21.3 (CKS1B), 1p32.3 (CDKN2C). Hybridization was performed according to the manufacturer’s instructions (Cytocell, Tarrytown, NY, USA).

### 2.3. Outcome Measures

The main endpoint was progression to multiple myeloma or amyloidosis. Progression to active disease was determined clinically by development of organ damage in the form of CRAB features, emergence of myeloma biomarkers, initiation of therapy (with or without CRAB features or biomarkers) or diagnosis of amyloidosis. TTP was calculated as the duration from diagnosis of SMM to the date of starting therapy. OS was defined as the time elapsed from diagnosis of SMM to death from any cause. Patients were censored in the TTP and OS analyses if they did not, respectively, progress or die at the date of last follow-up.

The reported BMPC percentage was determined by the highest BMPC percentage obtained by either bone marrow biopsy or bone marrow aspiration. Immunoparesis was defined as reduction in one or more of the uninvolved immunoglobulins below the lower limit of normal. In regards to cytogenetic analyses, patients were divided into two groups (the 2008–2014 group and the 2015–2020 group) to take into account that a plasma cell purifier was acquired in 2015 in our center. Thresholds for abnormal lactate dehydrogenase (LDH), albumin and β_2_-microglobulin were set according to references values at our center (≥250 UI/L, <35 g/L and ≥186 μmol/L, respectively).

### 2.4. Statistical Analysis

All the statistical analyses were done with IBM SPSS Statistics 26.0 and Excel 2016. A *p*-value < 0.05 was considered statistically significant for all analyses, unless specified otherwise.

Factors predicting progression were analyzed by Cox univariable and multivariable regression. The proportional hazards assumption was tested with the Schoenfeld residuals test. Survival analysis was performed using the Kaplan–Meier method. For the survival analysis, patients were assigned to one of three risk group, conforming to the new risk stratification model proposed by Mayo Clinic [14]. Namely, the patients were stratified according to three risk factors: BMPC ≥ 20%, FLCr ≥ 20 and M-protein ≥ 20 g/L. Patients without any of these risk factors were classified as low-risk, patients with one risk factor were classified as intermediate risk and patients with two or all risk factors were classified as high risk. Groups were compared with the two-tailed log-rank test.

## 3. Results

### 3.1. Patient Characteristics

We identified 113 patients, diagnosed between 2008 and 2020, who fulfilled the IMWG 2014 updated criteria for SMM. Among these patients, six were excluded because they had received chemotherapy for another cancer after the diagnosis of SMM and 18 because they had ≤2 years follow-ups or were missing critical data. Therefore, 89 patients were included in the study. Patient characteristics at diagnosis are shown in Table 2.

### 3.2. Prognostic Factors at Diagnosis

The median follow-up for the cohort was 51.0 months (interquartile range [IQR], 28.5–79.2) and the overall median TTP was not reached. In total, 31 patients progressed to symptomatic disease (multiple myeloma [*n* = 29], amyloidosis [*n* = 1] and plasma cell leukemia [*n* = 1]). Almost all patients (30/31, 96.8%) progressed within 5 years of diagnosis and approximatively half of the patients (16/31, 51.6%) within 2 years. At progression, the majority of patients (16/29, 55%) had anemia, while 34% (10/29) had renal failure, 24% (7/29) had bone lesions and only 7% (2/29) had hypercalcemia. In six patients, treatments were initiated because myeloma biomarkers thresholds were reached (namely, 3/6 had BMPC ≥ 60% and 3/6 had FLCr > 100).

In the univariable analysis, factors associated with increased risk of progression to symptomatic disease included a percentage of BMPC ≥ 20%, a FLCr ≥ 20, the light chain isotype, >1 M-protein spike and the presence of immunoparesis (Table 3). Sex, IgA isotype, intermediate and high-risk cytogenetic abnormalities, positive Bence-Jones, abnormal β_2_-microglobulin and MGUS prior to SMM did not result in an increased risk of progression. Although very close (*p* = 0.072), a serum M-protein > 20 g/L did not reach statistical significance either. A higher threshold for β_2_-microglobulin (>350 μmol/L, as suggested in other studies [28,29]) was also tested (data not shown), but did not result in a stronger correlation than the lower threshold (≥186 µmol/L: *p* = 0.970 vs. ≥350 µmol/L: *p* = 0.274). Abnormal albumin and LDH could not be included in univariable or multivariable analysis because no event occurred in either group.

The variables that reached statistical significance in the univariable analysis were included in the multivariable analysis, with the exception of FLCr. This risk factor had to be excluded because the higher proportion of missing data compromised other analyses. Although not being statistically significant in the univariable analysis, serum M-protein > 20 g/L was also included in the multivariable analysis because of the suspected impact as a prognostic factor based on other studies [14,15]. As illustrated in Table 3, light chain isotype and >1 M-protein spikes were no longer associated with an increased risk of progression in the multivariable analysis. In contrast, a percentage of BMPC ≥ 20% and immunoparesis remained statistically significant. Moreover, serum M-protein > 20 g/L reached statistical significance in the multivariable analysis, despite not quite reaching it in the univariable analysis.

### 3.3. Progression and Survival Analysis

**Figure 1:**
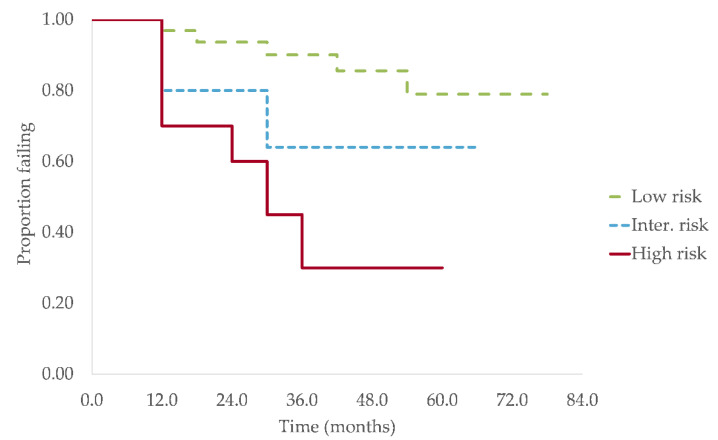
Progression free survival for the three risk groups according to the new Mayo Clinic model. Kaplan–Meier failure curves showing PFS for the low-risk group, intermediate-risk group and high-risk group. The estimated median TTP was not reached for the low and intermediate risk groups and was 29.1 months (95% C.I., 3.9–54.4) in the high-risk group (*p* = 0.006).

The median TTP for the entire cohort, the low-risk group and the intermediate-risk group were not reached and the estimated median TTP for high-risk group was 29.1 months (95% C.I., 3.9–54.4) (*p* = 0.006) (Figure 1).

The overall 1, 2 and 5 years PFS rate were, respectively, 87.6%, 81.6% and 60.8%. When subdivided into risk groups, the estimated proportion of progression-free patients at 1, 2 and 5 years were 96.8%, 93.4% and 77.5% for the low-risk group, 80.0%, 80.0% and 62.2% for the intermediate risk group and 70.0%, 58.3% and 29.2% for the high-risk group.

Overall, 25.8% (23/89) of our cohort died during follow-up. Only 39.1% (9/23) of those deaths were related to their gammopathy. The median age at death for the entire cohort was 80.0 years (range 62.5–89.4 years). In the subset of patient who died of a symptomatic gammopathy, the median age at death was 73.2 years (range 62.5–89.4 years) and in the subset of patient who died of unrelated cause, the median age at death was 81.3 years (range 66.9–87.2). The median overall survival (mOS) for the entire cohort was not reach. A subgroup analysis of each risk group revealed no significant difference in mOS (Low-risk vs. inter. risk HR [95% C.I.]: 2.021 [0.369–11.079] *p* = 0.418, low-risk vs. high-risk HR [95% C.I.]: 1.853 [0.338–10.156] *p* = 0.477).

## 4. Discussion

The purpose of our study was to test, in our population, the new risk stratification model proposed by the Mayo Clinic and to determine if similar results could be obtained in another cohort of SMM patients. Overall, our population matched well with previously described cohorts [3,4,14,15,16,17,18,19,20,21,22,23,24,25,26,27]. As illustrated in Table 2, almost all demographic and analytic parameters were close or within expected ranges. The median serum M-protein in our cohort was, however, somewhat lower than what had been described in the literature (11.7 g/L for our patients vs. 16.5–25 g/L [6,14,17,18] in other studies). This difference is potentially attributable to the recent changes in the diagnostic criteria of SMM. As mentioned earlier, SMM patients with BMPC ≥ 60%, FLCr ≥ 100 or > 1 bone lesion on MRI are now considered MM rather than SMM. We chose to exclude those patients from our cohort because their risk of progression has already been established and they usually start treatment at diagnosis. However, these asymptomatic patients were included in all earlier studies and in a majority of the more recent ones and this could explain, at least partially, why the median M-protein tend to be lower in our cohort. The fact that the median percentage of BMPC in our cohort was also on the lower side of reported values further supports this hypothesis. The remaining gap between our median M-protein and reported values in other SMM cohorts is perhaps attributable to a statistical fluctuation secondary to the small size of our sample.

In regard to external validity, approximately 15% of patients had to be excluded from our cohort because they had too few follow-ups or were missing critical data. The vast majority of those patients declined further investigations or follow-up because of debilitating comorbidities (advanced dementia being the most prevalent) that would have prohibited treatments if SMM were to progress to symptomatic disease. Consequently, excluded patients were likely sicker and older than those included in the cohort and this probably introduced some bias in our sample. However, this cohort is also more likely to be representative of the subset of patients who could benefit from a better stratification and early treatment. Therefore, we believe that excluding these patients does not compromise the external validity of our study.

Factors associated with increased risk of progression to symptomatic disease were a BMPC percentage > 20%, a serum M-protein > 20 g/L, a FLCr > 20 and immunoparesis. The data concerning the impact of immunoparesis on the risk of progression are inconsistent among studies. Indeed, in most studies, it appears to be an independent factor for progression [4,17,23,30,31] but in a more recent study with a large cohort, immunoparesis was not associated with an increase risk of progression [14]. In contrast, higher BMPC percentage, serum M-protein and FLCr, have consistently been associated with a higher risk of progression [4,14,15,23]. Among the variables presented in Table 3, we appear to be the first group to test the impact of MGUS prior SMM on the risk of progression. Before this study, we hypothesized that patients who had had a diagnosis of MGUS prior to their diagnosis of SMM would be more likely to progress to symptomatic disease (i.e., a sudden increase in BMPC percentage or serum M-protein could reflect the apparition of a more aggressive clonal plasma cell). However, our data showed no association between previous MGUS and the risk of progression. Nevertheless, further testing regarding the impact of this risk factor is necessary, since our follow-ups were undoubtedly too short to reach definitive conclusions and this variable was present only in a minority of patients, limiting the statistical power of our analyses.

Through the last decade, numerous cytogenetic abnormalities have been assessed by various research groups to identify those associated with an increased risk of progression. Many discrepancies remain in that field, but some abnormalities have been more consistently reported and are generally considered high-risk. Namely, the high-risk cytogenetic abnormalities are: t(4;14), del17p and gain 1q [22,24,32]. We explored the impact of t(4;14) and del17p on the risk of progression in our cohort and interestingly, found no correlation between those chromosomal aberration and progression. Concordant with previous studies, the incidence of those cytogenetic abnormalities were quite low in our cohort (7.7% for t(4;14) and 5.1% for del17p vs. reported incidence of approximately 10% and 5% [15,23,24], respectively). Consequently, only 5 patients had those high-risk cytogenetic abnormalities in our sample. It is, therefore, likely that our analyses lacked the statistical power needed to reveal a possible correlation between FISH status and progression. In one recent study with a large cohort however, del17p was determined not to be an independent factor for progression [15]. Perhaps our results reflected a true absence of correlation between del17p and progression, but more data is certainly essential to reach a definitive conclusion. Because no association between high-risk cytogenetic and progression was found, the alternative version of the 20/20/20 model proposed by Mateos et al. could not be tested in our cohort. As mentioned earlier, this group has recently suggested to include high-risk cytogenetic abnormalities as a fourth risk factor to the 20/20/20 model to increase its accuracy [15]. Further research, with a larger cohort and a more extensive FISH analysis, is thus necessary to test this alternative version of the 20/20/20 model in our population.

One limitation of our study was the relatively short follow-up (median of 51.0 months). Because of these shorter follow-up periods, the median TTP (mTTP) was reached only in the high-risk group. The mTTP in this group was 29.1 months, very close to the 29.2 months obtained by Lakshman et al. [14] for the same subset of patients. The fact that the mTTP was not reached in the low and intermediate groups is also consistent with data presented by Lakshman et al., given that the TTP in these groups were respectively 109.8 months and 67.8 months, which is longer than our median follow-up of 51.0 months. In an earlier study of SMM’s natural evolution, it was established that the overall risk of progression was 10% per year for the first 5 years after diagnosis [4]. We obtained similar results in our study, with overall PFS at 1, 2 and 5 years of, respectively, 87.6%, 81.6% and 60.8%. When subdivided according to the 20/20/20 model, the 2 years PFS for the low, intermediate and high-risk groups were 93.4%, 80.0% and 58.3% respectively. These results are also with very similar to data obtained by Lakshman et al. (PFS for the low, intermediate and high risk were 90.3%, 73.9% and 52.6% respectively) [14]. In another recent study, with a very large cohort of SMM patients [15], the 20/20/20 yielded nearly identical 2 years PFS (PFS for the low, intermediate and high risk were 93.8%, 82.1% and 55.8% respectively) [15].

Besides the relatively short follow-up, the other main limitation of our study was the small size of the cohort. The missing free light chain data from the earlier medical records contributed to further reduce the number of patients included in each risk group. As mentioned before, the small numbers of patients prohibited some analyses, such as cytogenetic status, and reduced the statistical power of others. As a result, most confidence intervals are quite large, but we believe the results shown here remain noteworthy because many analyses were statistically significant and most of the results correlate well with previous studies.

## 5. Conclusions

Despite some minor discrepancies, our cohort was overall similar to cohorts previously described in other studies and had adequate external validity. When Mayo Clinic’s new 20/20/20 risk stratification model was applied to our population, it adequately predicted the risk of progression to symptomatic disease at two years. As it relies on readily available biological parameters, this model is easy to use and can be applied in most clinical settings. In addition to the three factors used in the 20/20/20 model, we also found that immunoparesis was an independent factor associated with an increased risk of progression to symptomatic disease. Because some inconsistencies remain among different studies concerning immunoparesis, further investigations are necessary to elude the true value of this parameter in risk stratification. Finally, the impact of MGUS prior to the diagnosis of SMM on the risk of progression had not been tested until this study and our data showed no correlation between these two variables.

As early treatments of high-risk SMM remain controversial, a better understanding of risk factors associated with an increased risk of progression is essential to explore the impact of therapeutic options in this specific subgroup of patients. We believe our research is a step forward in the acceptance of the 20/20/20 model and thus, a step forward towards of a more unified definition of high-risk SMM.

## Figures and Tables

**Table 1:** Progression-free survival (PFS) at 2 years for SMM patients when stratified according to the 20/20/20 model with and without high-risk cytogenetic abnormalities.

Risk Stratification Model	Number of Risk Factors	2 Years PFS (%)
20/20/20 model (Lakshman et al.)		
Low risk	0	90.3
Intermediate risk	1	73.7
High risk	2–3	52.6
20/20/20 model with high-risk cytogenetics (Mateos et al.)		
Low risk	0	94.0
Low-intermediate risk	1	77.2
Intermediate risk	2	54.5
High risk	3–4	36.9

**Table 2:** Patients’ characteristics at diagnosis of SMM (*n* = 89) compared to reported values in other SMM cohorts [3,4,14,15,16,17,18,19,20,21,22,23,24,25,26,27].

Parameters	Study Cohort	Mayo Clinic Cohort	Other Cohorts ^1^
Age, years, median (range)	70.0 (39.0–86.9)	64.9 (30.2–92.1)	63–70 years [3,4,18,25]
Sex			
Male, *n* (%)	54 (60.7)	58.2%	53–64% [4,17,18]
Evolution of gammopathy			
MGUS prior to SMM, *n* (%)	21 (23.6)	n/d	28% [25]
New Mayo Clinic model markers			
BMPC (*n* = 89), %, median (range)	15.0 (7.5–50.0)	20 (5–50)	15–20% [14,16,17,18]
BMPC ≥ 20%, *n* (%)	21 (23.6)		
Serum M-protein (*n* = 89), g/L, median (range)	11.7 (0–35.2)	20 (0–50)	16–25 g/L [6,14,17,18]
M-protein ≥ 20 g/L, *n* (%)	8 (9.0)		
FLCr (*n* = 52), median (range)	7.4 (1.1–76.4)	7.8 (0.3–281.5)	6.9–11.6 [14,19,24]
FLCr ≥ 20, *n* (%)	14 (26.9)		
Heavy chain isotype (*n* = 89), *n* (%)			
IgG	52 (58.4)	75.8%	69–75% [4,14,17,18]
IgA	32 (36.0)	19.7%	19–31% [4,16,17,18]
IgM	0 (0.0)	0.9%	0.9–1% [14,16,19]
Light chain	5 (5.6)	3.6%	1–4% [14,16,17,19]
Nb of M-protein spike (*n* = 89), *n* (%)			
0	4 (4.5)	n/d	n/a
1	73 (82.0)	n/d	n/a
≥2	12 (13.5)	n/d	2–12% [19,21,26]
Immunoparesis (*n* = 86), *n* (%)			
Present	52 (60.5)	70.4%	50–83% [4,14,17,18]
Reduction of 1 Ig	17 (19.8)	n/d	18–31% [4,23,27]
Reduction of ≥ 2 Ig	35 (40.7)	n/d	29–52% [4,23,27]
Abnormal FISH (*n* = 54), *n* (%)			
2008–2014 (*n* = 15)	1 (6.7)	n/a	
2015–2020 (*n* = 39)	14 (35.9)	79.8%	
Deletion 17p	2 (5.1)	2.4%	1.7–6.1% [14,15,22,24]
Translocation t(4;14)	3 (7.7)	11.1%	8.9–11.1% [14,15,22,24]
Hyperdiploidy	8 (20.5)	43.4%	31.4–43.9% [14,15,22,24]
Other biomarkers, *n* (%)			
Positive Bence-Jones (*n* = 73)	33 (45.2)	n/d	22–40% [17,18,23]
LDH ≥ 250 UI/L (*n* = 63)	4 (6.3)	8.6%	n/a ^2^
Albumin < 35 g/L (*n* = 61)	3 (4.9)	30.8%	n/a ^2^
β_2_-microglobuline ≥ 186 μmol/L (*n* = 73)	46 (63.0)	n/d	n/a ^2^

Abbreviations: MGUS monoclonal gammopathy of undetermined significance, n/a not applicable, n/d not determined. ^1^ The ranges in this column represent the lowest and highest medians (or percentages) reported among other cohorts of SMM patients, not the actual range of values for a specific parameter reported in other studies. ^2^ Not enough available data to establish a range.

**Table 3:** Univariable and multivariable analysis of the impact of possible prognosis factors on TTP.

Possible Prognosis Factors	TTP, Months, Median (IQR)	Univariable Analysis		Multivariable Analysis	
		HR (95% C.I.)	*p* Value	HR (95% C.I.)	*p* Value
**Overall**	19.3 (9.73–35.6)	---	---	---	---
Sex					
Male	24.9 (11.0–40.3)	0.95 (0.46–1.96)	0.885	---	---
Female	18.0 (9.3–33.0)				
Evolution of gammopathy					
MGUS prior to SMM	18.0 (9.5–26.9)	1.61 (0.74–3.50)	0.231	---	---
No previous MGUS	24.5 (9.9–37.2)				
New Mayo Clinic model					
BMPC percentage					
BMPC ≥ 20%	13.9 (7.7–28.9)	3.91 (1.90–8.01)	**<0.001**	4.28 (1.90–9.61)	**<0.001**
BMPC < 20%	24.9 (14.9–43.9)				
Serum M-protein					
M-protein ≥ 20 g/L	7.5 (2.6–11.4)	2.64 (0.92–7.62)	0.072	4.20 (1.13–15.53)	**0.032**
M-protein < 20 g/L	24.9 (11.0–36.1)				
FLCr					
FLCr ≥ 20	14.9 (9.1–30.7)	3.25 (1.09–9.71)	**0.035**	---	---
FLCr < 20	21.4 (9.8–33.5)				
Heavy chain isotype					
IgG	24.9 (11.4–41.3)	0.91 (0.42–1.99)	0.815	---	---
IgA	18.5 (8.6–26.4)				
Light chain	19.2 (9.0–33.0)	2.42 (1.35–4.35)	**0.003**	1.46 (0.33–6.42)	0.619
Number of M-protein spike					
>1	18.0 (6.9–27.4)	2.39 (1.10–5.19)	**0.028**	2.40 (0.88–6.53)	0.086
1	24.5 (10.8–40.8)				
Immunoparesis					
Present	19.2 (9.6–28.9)	2.99 (1.27–7.03)	**0.012**	2.61 (1.07–6.41)	**0.036**
Absent	36.1 (19.0–47.6)				
FISH					
Normal and low-risk	21.9 (8.6–34.0)	1.57 (0.54–4.56)	0.403	---	---
Inter. and high-risk	18.0 (13.6–24.9)				
High-risk only	21.1 (11.4–25.3)	2.22 (0.71–6.96)	0.169	---	---
Biomarkers, *n* (%)					
Bence-Jones					
Positive	29.6 (9.9–39.2)	1.15 (0.52–2.53)	0.732	---	---
Negative	19.0 (10.0–38.2)				
β_2_-microglobuline					
≥186 μmol/L	18.5 (9.0–40.8)	0.98 (0.44–2.22)	0.970	---	---
<186 μmol/L	24.5 (10.7–39.8)				

Abbreviations: C.I.—confidence interval, IQR—interquartile range, HR—hazard ratio, TTP—time to progression. Values in bold indicate statistically significant *p*-values.

## Data Availability

The data that support the findings of this study are available from the corresponding author, upon request.
